# Effects of steroids on super-refractory status epilepticus in tick-borne meningoencephalitis

**DOI:** 10.1016/j.ebr.2024.100710

**Published:** 2024-09-16

**Authors:** Christine Heuer, Claudio Togni, Marian Galovic, Anna Czernuszenko, Giovanna Brandi, Ignazio de Trizio

**Affiliations:** a Institute for Intensive Care Medicine, University Hospital Zurich, Zurich, Switzerland; b Department of Neurology and Clinical Neuroscience Center, University Hospital and University of Zurich, Zurich, Switzerland; c University of Zurich, Zurich, Switzerland; d REHAB Basel, Clinic for Neurorehabilitation and Paraplegiology, Basel, Switzerland

**Keywords:** Status epilepticus, Tick-borne encephalitis, Steroids

## Abstract

We report a unique case of super-refractory status epilepticus (SRSE) secondary to tick-borne encephalitis (TBE) to evaluate the therapeutic challenges and potential benefits of steroid treatment in this context. A previously healthy 31-year-old woman was admitted to the hospital with fever, headache, vertigo, and meningismus, ultimately diagnosed with TBE. Despite empirical antimicrobial treatment, the patient’s condition deteriorated, leading to coma and SRSE. Various antiseizure medications and sedatives were administered without sustained success. Steroid treatment was initiated due to elevated intracranial pressure and persistent seizure activity. Following the administration of dexamethasone, electrographic status epilepticus resolved, though the patient developed clinical signs of increased intracranial pressure necessitating decompressive craniectomy. The patient’s condition stabilized with a combination of antiseizure medicazions. Despite cessation of SRSE, the patient remained in a minimally conscious state at discharge, showing only gradual improvement over time. The use of steroids in TBE is controversial, with limited reports of potential benefits. In this case, steroid administration coincided with the cessation of SRSE, and authors explore its potential benefit considering its immunomodulatory effects.

## Highlights

• Super-refractory status epilepticus is a complication of tick-borne encephalitis.
• Use of steroids is controversial in TBE.
• In our case steroids had a potential role in cessation of the status epilepticus.

## 1 Case presentation

A previously healthy 31-year-old woman presented to the emergency unit of an external hospital, complaining of persisting fever, headache, vertigo and meningismus for five days and presenting meningismus without neurological deficits. Lumbar puncture showed a pleocytosis of 47 mononuclear cells/µl and serum was positive for IgM against TBE virus, but negative for anti-TBE IgG. No tick bites were observed clinically nor reported in the medical history. She had not been TBE-vaccinated before. Empirical antimicrobial treatment with acyclovir, ceftriaxone and dexamethasone was stopped after viral and bacterial PCR assays, Gram stain and microbiological cultures of cerebrospinal fluid resulted negative.

Three days after admission (day 8 after symptom onset), the patient developed coma requiring orotracheal intubation and was transferred to the intensive care unit (ICU). The first computed tomography (CT) of the head revealed no pathological findings and the patient was empirically started on levetiracetam (1000 mg i.v. bid) assuming an epileptic cause of the coma. Serologic analysis of the same day showed a seroconversion of anti-TBE IgG with increasing anti-TBE IgM confirming the diagnosis of TBE within 72 h from the first medical contact. The cerebral MRI on the following day showed TBE typical changes and the routinely recorded EEG displayed an ictal-interictal continuum with generalized periodic discharges with a frequency below 2 Hz, responsive to midazolam. Fulfilling the criteria for a possible non convulsive status epilepticus (NCSE), levetiracetam dosage was increased to 1250 mg i.v. bid, and a continuous midazolam infusion was started (up to 5 mg/h). On day 10, the EEG showed a variable delta-theta-alpha curve with repeated generalized rhythmic delta activity with a frequency below 2 Hz and centrally located epileptic discharges, which only rarely generalized. Levetiracetam was further increased to 1500 mg i.v. bid, and sodium valproate 300 mg i.v. qid was added. In the following night, the patient presented left sided cloni responsive to an increase in the continuous midazolam infusion up to 20 mg/h. The following days EEG displayed generalized spike wave and polyspike wave complexes with a frequency of 1.5–2.5 Hz responsive to midazolam and propofol. NCSE was diagnosed by EEG and clinical criteria and the patient was transferred to our neurocritical care unit for continuous EEG monitoring.

**Fig. 1:**
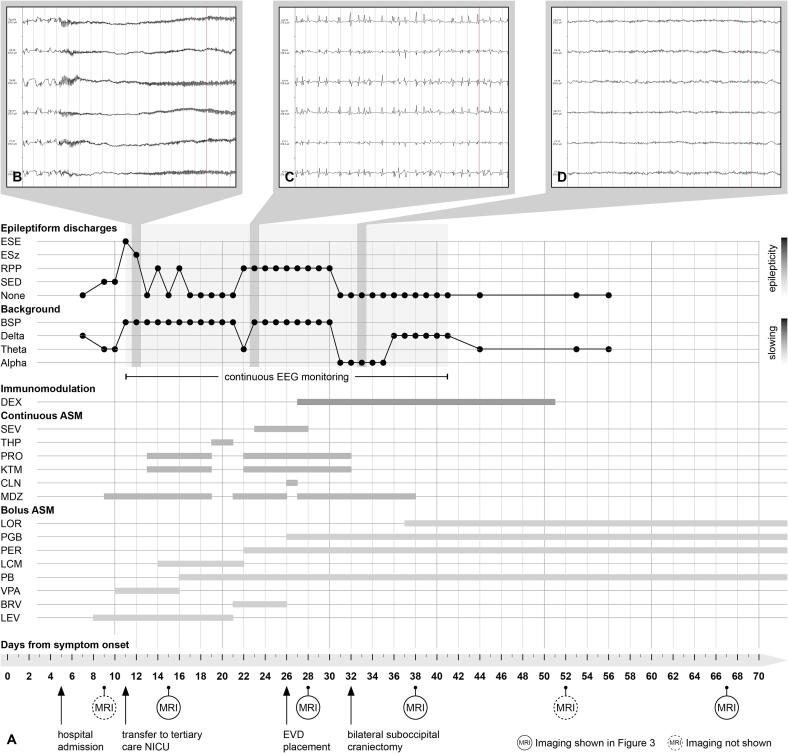
Graphical illustration of disease course. A) Intermittent and continuous EEG findings were simplified and categorized according to the two dimensions “epilepticity“ and “generalized slowing“ (upper part) in order to convey potential effects of pharmacological and surgical interventions (middle and lower part). Timepoints of serial MRI findings as shown in Fig. 3 are noted (lower part). Representative EEG snippets are shown as inlays (Fig. 1B, C and D) and as full-size images in Fig. 2. B) Generalized electrographic seizure as noted on continuous EEG on day 12 despite high-dose midazolam IV. C) Ictal-interictal continuum (2 Hz generalized periodic discharges) as noted on continuous EEG on day 24 despite sevoflurane narcosis and achievement of intermittent burst-suppression pattern. D) Alpha-beta background activity without epileptiform discharges as noted on continuous EEG on day 33 while tapering midazolam IV. Note the sudden disappearance of epileptiform findings on day 32. ASM, anti-seizure medication; BRV, brivaracetam; BSP, burst-suppression pattern; CLN, clonazepam; DEX, dexamethasone; EEG, electroencephalogram; ESE, electrographical status epileptics; ESz, electrographical seizure; EVD, external ventricular drain; IV, intravenous; KTM, ketamine; LCM, lacosamide; LEV, levetiracetam; LOR, lorazepam; MDZ, midazolam; MRI, magnetic resonance imaging; PB, phenobarbital; PER, perampanel; PGB, pregabalin; PO, per os; PRO, propofol; RPP, rhythmic or periodic pattern; SED, sporadic epileptiform discharges; SEV, sevoflurane; THP, thiopental; VPA, valproic acid.

**Fig. 2:**
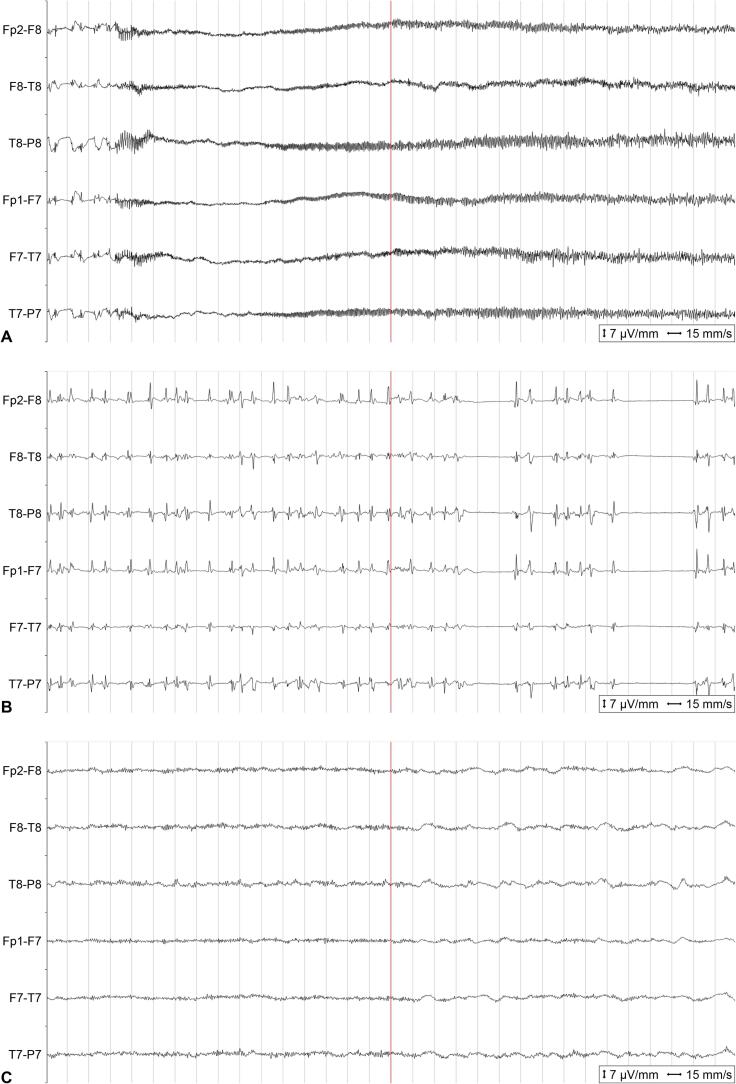
EEG findings. The snippets correspond to the inlays in Fig. 1. (A) EEG on day 12. Generalized onset of spike-wave-activity evolving into high-frequency spiky discharges of waxing and waning amplitude consistent with a generalized onset electrographic seizure. This was recorded while on high-dose midazolam IV. (B) EEG on day 24. Spiky and sharp generalized periodic discharges of fluctuating frequency averaging 1.5–2.0 Hz corresponding to a pattern on the ictal-interictal continuum. This was recorded during sevoflurane narcosis. (C) EEG on day 33. Symmetrical alpha-theta activity without any apparent antero-posterior gradient without any epileptiform discharges. This was recorded while tapering midazolam.

**Fig. 3:**
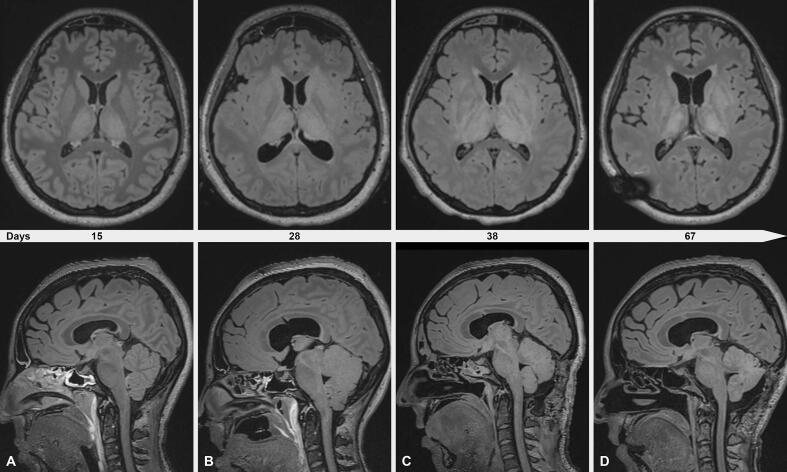
Longitudinal magnetic resonance imaging. For comparability, all transverse sections were taken at the level of the splenium corpori callosi and all sagittal sections were taken slightly lateral to the midline. A) Bilateral mesodiencephalic inflammatory T2/FLAIR-hyperintensities typical of TBE on day 15. B) Progression of the aforementioned findings on day 28. Furthermore, newly developed hydrocephalus despite placement of an external ventricular drain and generalized infratentorial edema. C) Further progression of the aforementioned findings on day 38. Regression of the infratentorial edema after bilateral suboccipital craniectomy in the meantime. D) Regression of supra- and infratentorial inflammatory findings on day 67. Meanwhile, a ventriculoperitoneal shunt was placed (note the artifact in the right parietal region).

The NCSE was treated in the following weeks with multiple combinations of antiseizure medications (ASM) including lacosamide, brivaracetam, phenobarbital, pregabalin and perampanel, as well as continuous infusion of clonazepam, ketamine, propofol, thiopental, and the inhaled anaesthetic sevoflurane (Fig. 1a). Despite this, epileptic activity with generalized periodic discharges at a frequency of 2 Hz (Fig. 1c, Fig. 2b) could only be suppressed for short period of time and rebounded after reduction of the medications, thus constituting a SRSE. Treatment with steroids or immunosuppressants, as otherwise recommended for new-onset refractory status epilepticus (NORSE)[1], was repeatedly discussed and was not recommended by the neuroimmunologists due to concerns for potential worsening of TBE. Screening for anti-neuronal antibodies returned negative. The MRI on day 15 showed constant changes typical of TBE (Fig. 3a).

Ten days later (on day 26), due to elevated intracranial pressure (ICP) measured during a diagnostic lumbar puncture, a head-CT scan was performed and revealed cerebral edema and tonsillar herniation. An external ventricular drain (EVD) was inserted, and intravenous dexamethasone 8 mg bid was started to treat both the cerebral edema and suppress the inflammation that could represent a trigger for the SRSE. Three days after starting dexamethasone, a complete resolution of generalized periodic discharges was documented by EEG and continuous background activity emerged (Fig. 1d, Fig. 2c). Despite the cessation of SE, the patient showed progressive clinical signs of increased ICP with pupillary abnormalities, necessitating a decompressive craniectomy of the posterior fossa. After that, propofol, ketamine and midazolam could be stopped stepwise in short course (Fig. 1) and the patient remained without epileptic activity under a combination of ASMs (phenorbarbital, perampanel, and pregabalin). After 10 days, dexamethasone was tapered by 2 mg every two days until discontinuation. Despite the cessation of SE, the patient remained in a minimally conscious state at ICU discharge, opening her eyes when approached, smiling, and showing minimal reactions to external stimuli.

The patient was subsequently transferred to a rehabilitation clinic. As the minimally conscious state persisted, another lumbar puncture was performed which displayed a normalization of the cell count with persistent disrupted blood-cerebrospinal fluid barrier function. Assuming a TBE persistence because of persistent IgM in serum and CSF as well as intrathecal IgG synthesis, the patient was treated with a course of intravenous immunoglobulins (IVIG) over five days (cumulative 90 g), four months after disease onset. Under unchanged ASMs, a further improvement of background activity up to alpha–beta mixed frequency was observed in the EEG with persistence of isolated epileptic discharges. At discharge, the patient was able to show an adequate emotional response and to communicate intermittently by lid closure.

## 2 Methods

We report the case of a single patient admitted to the neurocritical care unit of the Zurich University Hospital. A written informed consent for disclosure of any recognizable personal information and for the publication of this report and accompanying images was obtained from the patient’s legal representative. The study complies with the Declaration of Helsinki, the Guidelines on Good Clinical Practice (GCP-Directive) issued by the European Medicines Agency as well as with Swiss law and regulatory authority requirements. This article was prepared following the CARE Guidelines [2].

## 3 Discussion

TBE is an infectious disease caused by the TBE-virus (*Flavivirus* genus, family *Flaviviridae*). The European subtype is transmitted by an infected castor bean (*Ixodes Ricinus*) tick and is endemic in rural and forested areas of central, eastern and northern Europe. Different meningoencephalitis symptoms can be observed in about 40 % of adult patients [3].

We report a case of SRSE due to TBE that may have responded to steroids. According to the current recommendations for treatment of SE [4] we set up a stepwise therapeutic approach. A lasting interruption of SE was not achieved with these measures.

The use of steroids in TBE is controversial and only a few cases have reported a possibly beneficial effect in TBE [5], [6]. The consensus review of the European Academy of Neurology on the management of TBE do not recommend the use of corticosteroids as immunomodulating therapy due to lack of evidence and the potential risk of adverse effects [3].

On the other hand, in a similar condition to that of the patient, as NORSE, immunotherapy, such as corticosteroids, rituximab, anakinra or tocilizumab, is recommended as a second-line treatment by international consensus guidelines for the management of NORSE [1], [7], [8]. Even though there is no high-quality evidence supporting the effectiveness of immunotherapy in NORSE, these recommendations reinforce the concept of immune mechanism underlying NORSE [1], [9]. In the presented case, the introduction of corticosteroid therapy was followed by a sustained control of epileptic activity. Although a causal role of steroids in the improvement of SE cannot be definitely confirmed, the rapid cessation of SE following the administration of steroids suggests a potential impact of steroids on the termination of sustained epileptic activity in our patient.

In particular in the presented case of SRSE, since corticosteroid therapy was also given to treat cerebral edema, it is difficult to define whether the improvement in epileptic activity was attributable to the anti-inflammatory effect or to the improvement in cerebral edema and thus also to the placement of the EVD and to the suboccipital craniectomy. The patient received IVIG 4 months after disease onset. IVIG is also recommended as an early treatment in NORSE [7] and there are case reports on the successful treatment of arboviral encephalitis with IVIG, especially when treated early [10]. Whether the further clinical and electroencephalographic improvement is due to the anti-inflammatory effects of IVIG remains undetermined.

## 4 Conclusion

In the context of TBE complicated by SRSE, it is not possible to define a consensus or evidence-based treatments and the feasibility of large studies is very limited. For this reason, we believe it is fundamental to share our experience suggesting a possible role of corticosteroids both as an adjuvant treatment worthy of further investigation in the context of SRSE as well as a possible immunomodulatory therapy for the TBE.

## Funding

No funding was received for this study.

## Ethical statement

A written informed consent for disclosure of any recognizable personal information and for the publication of this report and accompanying images was obtained from the patient’s legal representative. The study complies with the Declaration of Helsinki, the Guidelines on Good Clinical Practice (GCP-Directive) issued by the European Medicines Agency as well as with Swiss law and regulatory authority requirements.

## 7 Authors’ contribution

CH, IDT had the idea for the case report and wrote the manuscript, CT created the figures. GB, MG lead the clinical management of the patient. GB supervised the editing of the manuscript. MG, AC, GB gave critical input to the manuscript.

## CRediT authorship contribution statement

**Christine Heuer:** Writing – original draft, Conceptualization. **Claudio Togni:** Writing – review & editing, Visualization, Data curation, Conceptualization. **Marian Galovic:** Supervision. **Anna Czernuszenko:** Supervision. **Giovanna Brandi:** Writing – review & editing, Supervision, Project administration, Data curation, Conceptualization. **Ignazio de Trizio:** Writing – review & editing, Writing – original draft, Supervision, Data curation, Conceptualization.

## Declaration of competing interest

The authors declare that they have no known competing financial interests or personal relationships that could have appeared to influence the work reported in this paper.
